# Exploring Paternal Mentalization Among Fathers of Toddlers Through a Clay-Sculpting Task

**DOI:** 10.3389/fpsyg.2021.518480

**Published:** 2021-03-02

**Authors:** Nehama Grenimann Bauch, Michal Bat Or

**Affiliations:** ^1^College of Business, Arts and Social Sciences, Brunel University London, Uxbridge, United Kingdom; ^2^Emili Sagol Creative Arts Therapies Research Center, Faculty of Social Welfare and Health Sciences, University of Haifa, Haifa, Israel; ^3^The School of Creative Arts Therapies, Faculty of Social Welfare and Health Sciences, University of Haifa, Haifa, Israel

**Keywords:** mentalization, fathers, toddlers, paternal mentalization, parental mentalization, sculpting, clay, parenting

## Abstract

This study explored parental mentalization processes as they unfolded during a sculpting task administered to fathers of toddlers. Parental mentalization—the parent’s ability to understand behavior (his/her own as a parent and that of their child) based on its underlying mental states (Luyten et al., 2017)—is considered crucial within parent–child relationships (Fonagy et al., 1998) and child development (Steele and Steele, 2008). Eleven Israeli first-time fathers (*n* = 11) of children aged 2–3 (mean = 2.3) were asked to sculpt a representation of themselves with their child using clay. Following the task, the fathers were interviewed while observing the sculpture they had created. Qualitative thematic analysis integrated three types of data—video footage of the sculpting processes, the sculptures themselves, and the transcripts of the post-sculpting interviews. By focusing on data extracts relating to mentalization processes, three main aspects of the clay-sculpting task and interview were identified as processes that either preceded controlled mentalization instances and/or related to their underlying dynamics: (1) discussing the sculpting process elicited the father’s curiosity and wonder; (2) observing the sculpture/sculpting process revealed gaps in paternal representations; and (3) the preplanning of the sculptures sparked non-verbal exploration of metaphors and symbolism. Special attention was given, in the analysis, to the interplay between verbal and non-verbal aspects of mentalization as they appeared in the metaphorical representations that arose through the sculpting process. Comparing this sample to a previous sample of mothers who were given the same task, similarities and differences were explored, with specific reference to topics of embodiment, gender roles, paternity leave, and an active approach in art therapy. The discussion indicates that clay sculpting may offer unique insight into implicit parental mentalization. Possible clinical applications are discussed, with reference to attachment theory and clinical art therapy approaches.

## Introduction

Parental mentalization—parents’ ability to understand and reflect upon the mental states that lie beneath their child’s and their own behaviors—is a concept that has been researched extensively (Allen et al., 2008; Sharp and Fonagy, 2008). It has been linked, among other factors, to the child’s attachment security (Fonagy et al., 1998; Schechter et al., 2005) and social-cognitive development (Sharp and Fonagy, 2008). Research has focused specifically on maternal mentalization and mind-mindedness, i.e., the mother’s ability to see her child as a mental agent and to use mental state terms in her narratives (e.g., Slade, 2005; Schiborr et al., 2013). Although an individual is born with an innate disposition to develop mentalizing capacities, parental reflective functioning (RF), the parent’s capacity to mentalize about their children and themselves as parents, is crucial for a child to develop mentalizing capacities (Slade, 2005). This is especially true during the first 6 years of life, as the child gradually passes critical developmental milestones (see Fonagy et al., 2002; Allen et al., 2008). Research has focused mainly on maternal mentalization; paternal mentalization has yet to be fully explored.

Allen et al. (2008) distinguish between explicit and implicit mentalization. Explicit or controlled mentalization is a verbal narrative or symbolic form of relatively conscious reflection requiring intentional cognitive effort. By contrast, implicit or automatic mentalization requires a less controlled and declarative effort; though not necessarily unconscious, it is intuitive and unreflective. We mentalize implicitly mainly during interpersonal interactions (Allen et al., 2008) when we are required to react quickly. Based on neurobiological studies, Luyten and Fonagy (2015) defined three mentalizing polarities: (1) internal mentalizing, which focuses on internal features and involves more active and controlled reflection, versus external mentalizing, which focuses on external features such as a smile or a physical gesture; (2) self-mentalizing, which involves understanding one’s own mental states, versus mentalizing that involves understanding the mental states of the other; (3) cognitive mentalizing, which relates to someone’s thinking—their desires, beliefs, and reasoning—as opposed to affective mentalizing, which relates to feelings—to identifying, modulating, and expressing emotions. These mentalization polarities are complexly linked and interrelated. For example, empathizing links affective and automatic mentalization; partially implicit in nature (Behrends et al., 2012), it stems from a mirroring, less conscious emotional response to others. To summarize, mentalization is an intricate multicomponent process (Fonagy and Allison, 2012), and its assessment requires a sensitive and multifaceted approach.

Methods for the assessment of parental RF, such as the Parent Development Interview (PDI) (Slade et al., 2004), are predominantly based on parents’ verbal descriptions of their relationship with their child. Interventions to facilitate mentalization are also predominantly verbal, including such techniques as reflective mentalizing questions about the child (e.g., Suchman et al., 2017) and parental group mentalizing sessions (Slade, 2007; Baradon et al., 2008). Within clinical settings, four main therapeutic interventions appear to facilitate and enhance parental mentalization (Slade, 2007). In order to represent the child’s mental states to the parent, the clinician may: (a) continuously model reflectiveness; (b) facilitate wonder by enhancing curiosity about the child’s experience; (c) elicit affect by talking about concrete situations, enhancing mentalization by reflecting on “hot” unregulated moments (which include implicit mentalization); and (d) hold the parent in their own mind, enabling access to childhood memories too painful to remember, since memories based on non-verbal experiences often cannot be accessed through verbal paths alone.

Attachment is transmitted intergenerationally (Lyons-Ruth, 1998; Shai and Belsky, 2011) via the implicit process by which the parent’s representations of early experiences with their own parents, combined with daily parent–child interactions (Dollberg et al., 2010), are eventually internalized by the child. Bowlby (1969) called this “the internal working model mechanism.” Fonagy and Target (2007) coined a new term, “embodied cognition,” to emphasize the significance of bodily sensations in addition to cognition-based internalization in forming the attachment behaviors on which parental symbolic representations are founded. Therefore, many forms of arts, which induce embodied experiences, may tap into memories of physical sensations, auditory, kinesthetic, and symbolic representations and access preverbal, physical, and implicit representations (Verfaille, 2016). This is also true within the realm of arts-based parental representations (Bat Or, 2010; Bat Or and Grenimann Bauch, 2017). In art therapy, by using artistic expression within the context of a therapeutic relationship (Rubin, 1999), reflecting on such experiences serves to facilitate personal growth and change.

Central therapeutic features of clay work, such as revealing unconscious materials, concretizing, symbolization, and facilitating verbal communication, can contribute to a significant exploration of self and other (Sholt and Gavron, 2006). Symbolizing uses a mark or character as a conventional representation of an object (Oxford Dictionary, 2019), while a metaphor, according to Lakoff and Johnson (1999), “allows conventional mental imagery from sensorimotor domains to be used for domains of subjective experience” (p. 45). The body–mind bottom-up hierarchical approach, based on the expressive therapies continuum (ETC) model (Hinz, 2009; Lusebrink and Hinz, 2016) gradually moves from the somatosensory aspects of clay work to making meaning on a metaphorical and symbolic level (Nan and Ho, 2017). Self-expression through touch and movement may elicit primary modes of communication and trigger implicit memories of touch (Souter-Anderson, 2010; Elbrecht, 2012). This may be observed across different artistic media, such as drawing, collage-making, or creating with found objects (Moon, 2010). Specifically, the plasticity of clay enables a dynamic search for a desired expression, while its multidimensional aspect may unearth multiple meanings. Within the clay work, the move toward metaphorical thinking allows for an exploration of the creator’s inner world from different, fresh, and novel perspectives (Henley, 2002; Sholt and Gavron, 2006).

Bat Or’s qualitative study found that clay sculpting of mother and child figures facilitated mentalization among Israeli mothers (Bat Or, 2010). Four specific characteristics of the task were found to elicit parental mentalization: visual reflectiveness, wondering, transformation, and implicit memories. Case studies have suggested that when an affect is represented in concrete terms—such as an absence of legs representing feelings of immobility—the therapist’s active curiosity and questioning may help link the visual experience to an emotional one (Havsteen-Franklin, 2016a; Verfaille, 2016). These qualities of clay work may be beneficial when developing much needed specific father-based assessments and interventions (Scott and Crooks, 2007; Bureau et al., 2016)—a need stemming from a shift in Western societal norms regarding paternal roles (Wheelock, 1990) and the concurrent increase in positive paternal engagement (Pleck, 2010).

Father engagement has been found to have a positive effect on children’s social, behavioral, psychological, and cognitive outcomes (Sarkadi et al., 2007). Specifically, paternal RF appears crucial to children’s socioemotional development (Benbassat and Priel, 2015). Accordingly, fathers who possess greater mind-mindedness capacities have children who demonstrate higher theory of mind performance (Lundy, 2013). In addition, higher levels of paternal sensitivity are associated with mentalizing capabilities (Haßelbeck, 2014) and, subsequently, more infant–father attachment security (Lucassen et al., 2011). Paternal RF levels appear to influence the development of child anxiety (Esbjørn et al., 2013).

Studies of paternal brain activity suggest a neurobiological adaptation process in the first 2 years of the child’s life, supporting the idea of a gradual attachment-building process for fathers, while mothers show similar attachment brain activities earlier on (Mascaro et al., 2013). Tharner et al. (2016) also suggest that past caregiving experiences and personal involvement with the child affect a father’s mentalizing capabilities and behavior when interacting with his child.

Studies of father-focused mentalizing and subsequent early intervention models are scarce (e.g., Lawrence et al., 2012; Söderström and Skårderud, 2013), and only a few include non-verbal input as well, such as video feedback of parent–child interactions (Lawrence et al., 2012). The present study is part of a qualitative study that used a clay-sculpting task to inquire into the parental representations of fathers of toddlers. Four main paternal representations were observed: encouraging the child’s independence versus protecting, movement and playfulness, using abstraction and metaphors, and being satisfied with imperfections. Focusing on instances of mentalizing as they arose during and after the clay task, we asked: What processes preceded the appearance of paternal mentalization, and what was their nature? What are the underlying dynamics of controlled paternal mentalization as it relates to the non-verbal aspects of three-dimensional creative processes?

## Materials and Methods

### Participants

The non-clinical sample included 11 Jewish Israeli married fathers, each with a first and normative child aged 2–3 years old (mean = 2.3). Six of the children were boys and five were girls, for gender balance. The fathers’ ages ranged between 30 and 37 (mean = 33.8). Most of the fathers (72%) rated themselves as having an above-average income and level of education. Specific information can be found in Table 1. All fathers rated themselves as very involved during the pregnancy and birth. Only one father had previous experience sculpting with clay.^1^ Participants were recruited by purposeful sampling (Patton, 2002), such as announcements in playgrounds and kindergartens, or social media outreach to parents. This was carried out concurrently with data collection and analysis. The interviews were held during the summer of 2014 when most of the region was affected by geopolitical events; the participants all lived in areas that experienced sirens, rocket attacks, and calls to military reserve duty. Possible effects of these events on the study are discussed toward the end of this paper.

**TABLE 1:** General demographic data—age, partner’s employment, and paternity leave^1^.

Father	Age	Child gender		Child age	Paternity leave			Partner’s working status	
1	36	Male		2.5	1 month			Full-time employee	
2	30	Female		2.5	Circa 20 days			Part-time employee	
3	36	Male		2	2 weeks			Unemployed	
4	33	Male		3	7 days			Full-time employee	
5	32	Female		2	2 months			Full-time employee	
6	37	Female		3.3	5 days			Part-time employee/self-employed	
7	31	Male		2	1 week			Self-employed	
8	36	Male		2	4 days			Part-time employee	
9	36	Male		2.2	5 days			Full-time employee	
10	35	Female		2	2 days			Full-time employee	
11	30	Female		2.5	2 days a week for 6 months [calculated as 60 days]			Full-time employee	
Summary of age and paternity leave									
Fathers’ average age:		33.8	Children’s average age:		2.3	Paternity leave average:		19.5 days

*^1^Additional data can be found in the Supplementary Table 8.*

### Procedures

The study was approved by the local academic ethics committee, and participants signed informed consent forms. Individual meetings were held with the fathers in their homes and included a sculpting task and a sculpting interview. These were not limited in time and lasted between 40 min and an hour and a half.^2^ Each father was given a single round 15 cm × 15 cm lump of clay placed on a mobile wood surface with five wooden sculpting tools nearby. At the warm-up stage, the researcher provided a few basic instructions about creating three-dimensional sculptures with clay. The participants were then told: “Please make a three-dimensional sculpture of yourself with your child, or the relationship between you and your child.” The sculpting process was videotaped from the approximate angle of the fathers’ sight, focusing on their hands.

### The Interview

The sculpting process was followed by a semistructured interview (Patton, 2002) constructed for the purpose of this study and based on Bat Or’s (2010) study with mothers, with specific modifications for fathers. The interview included the following components: observation of the sculpture from all angles by slowly rotating the sculpture in front of the participant; elicitation of the participant’s subjective understanding, by asking, for example, “What do you see?” (Betensky, 1995); verbal reconstruction of the sculpting process, by asking, for example, “Do you remember whether you experienced obstacles or frustrations during the sculpting process, and if so, at what point in the process?”; a few projective questions regarding the clay figures, for example, “If the father figure could talk, what would it say?”; and the elicitation of possible resemblances or differences between the sculpture and the image the participant has of himself as a child with his own father. Additionally, fathers were given time to comment and share their thoughts about fatherhood and about the influence of the interviewer’s gender and profession.

### Data Analysis

**FIGURE 1:**
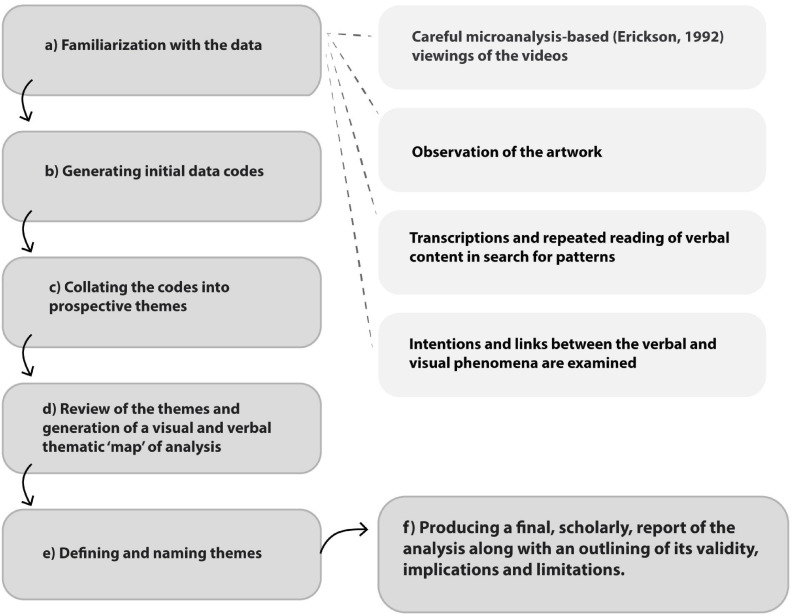
Phases of thematic analysis.

Data included the sculpting process, the sculptures, and the postsculpting interviews. A thematic analysis approach was used, with the aim of discerning, examining, and outlining patterns within the entire body of visual and verbal data (Braun and Clarke, 2006), as described in Figure 1. A primary theoretical approach was used to focus particularly on data extracts relating to mentalization and RF (based on Slade, 2005; Allen et al., 2008) and specifically on the processes that may have fostered the appearance of controlled mentalization. An underlying contextual approach (Carson and Samura, 1997) attempted to acknowledge the way the individual fathers make meaning of their experiences while also considering the influence of the broader social backdrop. The content of the data was examined at a latent level—through a conceptual mentalization lens—which involved interpretive work during the development of themes.

The process of interpreting both the observable and the unobservable (for example, the mentalizing underlying a metaphor that a participant described) was inspired by the hermeneutic circle which embraces the subjectivity of the interpreter (Ricoeur, 1981) while trying to stay as true as possible to the subjects’ own descriptions. To create an embodied bracketing and meta-reflection process (Luca, 2009), the researchers and authors reflected consciously on their perspectives as women therapists with prior knowledge. A specific consideration was given to the researchers’ identities as mothers and potential related biases were discussed. The thematic analysis consisted of six phases, as described in the following diagram.

The primary purpose of the study was to explore the nature of paternal mentalization and the processes that preceded its appearance within the context of the fathers describing their experiences through non-verbal and verbal means. Therefore, there was no overall classification of the fathers’ mentalizing capacities; the study included only detection and analysis of controlled mentalizing instances relating to fatherhood and the processes that appeared to foster or hinder their appearance. Concurrent to the coding of themes related to paternal representations, mentalization was detected in the verbal data, for example, when the father interpreted his sculpting process or sculpture contents in mental terms and spoke of mental states underlying his behavior and that of his significant others (his child or partner). Non-verbalized mentalization was also detected in metaphors and symbols that arose, including specific content that involved reflection on mental states, such as the child’s or father’s wishes, intentions, desires, thoughts, or feelings. These data extracts were coded and organized, taking into consideration Luyten and Fonagy’s (2015) four mentalizing polarities (controlled/automatic, self/other, cognitive/affective, and external/internal). Switches in mentalization, a rapid shifting from controlled to automatic thinking (Fonagy and Luyten, 2009), were found when the participant: (1) suddenly changed the topic of the discussion (e.g., offered remarks which revealed curiosity or questioned his own underlying intentions and mental states when making his sculpting choices, but then gave external explanations or discontinued the exploration of mental content); (2) generalized or used non-mentalizing words such as “just,” “always,” “never,” “it’s nothing important,” and similar expressions (Allen et al., 2008). Salient verbal and non-verbal mentalization data were sorted and compared in tables according to the prompts, probes, and/or events that preceded them and a second reviewer assessed them for reliability purposes. The final data was synthesized and analyzed relating back to the relevant literature. The names of the fathers and children have been changed to ensure confidentiality. All interview excerpts were translated from Hebrew by the researchers and are rendered in language as close as possible to the source regarding grammar and word use.

## Findings

In all 11 interviews, instances of controlled parental mentalizing were observed, either verbally or visually (within the metaphorical content of the sculpture). Almost all the verbal evidence of explicit parental mentalization (95%) was found in the interview transcripts as opposed to the sculpting process; only in two cases, verbal indications of controlled mentalizing also appeared during the sculpting process. The *Findings* section outlines three main themes that relate to the context within which the instances of paternal mentalization appeared: (1) discussing the sculpting process elicited curiosity and wonder; (2) observing the sculpture/sculpting process revealed gaps in paternal representations (for example, explicit vs. implicit); and (3) the preplanning of the sculptures sparked non-verbal exploration of metaphors and symbolism.

### Discussing the Sculpting Process Elicited Curiosity and Wonder

In seven of the sculpting interviews (63%), indications of mentalizing appeared in response to sculpting process questions, whereas in only two instances (18%) was mentalizing evidence noted in reaction to the visually reflective question “What do you see?” Table 2 presents the prompt, probe, or event preceding mentalizing instances during the interviews.

**TABLE 2:** Prompt, probe, or event preceding mentalizing instances during the interviews.

Prompt, probe, or preceding event	Number of indications of verbal mentalization*
Process-related questions (describing the sculpting process or describing feelings and thoughts during the process)	7
The sculpture was used as a reflective tool (the sculpture/process reflected a gap between wishes/fantasies and reality)	6
“Did you learn something new?”	4
“What would the figures say, if they could speak?”	3
At the end of the interview, after being asked how they feel about the interviewer taking the sculpture	3
Intergenerational transmission-related questions	2
“What do you see?”	2
When asked about possible differences between mothers and fathers	1

*Representing the number of fathers who mentalized following a specific prompt or probe. The numbers do not represent specific RF types or frequencies.

**FIGURE 2:**
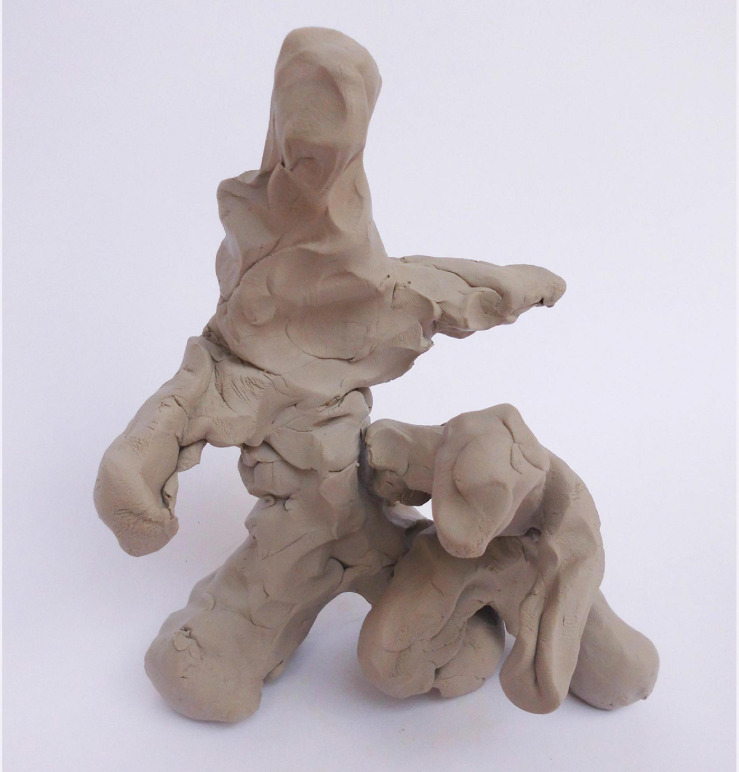
Nachshon’s sculpture.

Among the seven fathers who mentalized in response to sculpting-process-related questions was Nachshon, the father of Dekel, a boy aged 26 months. Nachshon quickly sculpted two very simplified human figures of father and son, sculpting first the father figure and then the son (Figure 2). He then spent circa 2 min moving the child figure closer to the father, changing and fine-tuning the figure’s positions and gestures. As he described the process, he explained his movements and spoke of distance and closeness, which led to a more general reflection about himself as a father, his son’s experience, and his own feelings:

Nachshon: The first instinct is that I’m not there enough, that I’m not attentive enough to him, so he’s, like, behind me. I’m very occupied with myself… but if I think of it, taking a second, gentler view, then I do see him. I just… I allow the distance at which he walks. I’m not necessarily with him, or face to face (yes). I’m, in this way, around, from a distance. But I think that he does feel me. He leans on me with his step forward (interviewer: he is connected to you) {both interviewer and Nachshon looking at the connection part of the sculpture} yes, he is connected to me. Very sad, very sad…

Nachshon’s exploration of the sculpting process led him to consider the gap between his first intuitive impression of himself as a father and his later reflective, “gentler” look at himself. He went on to describe feelings of sadness and guilt at not being present enough and missing out. “Some sort of feeling, also, that my son is moving forward, walking into the world.” Through physical movement and slight changing of the sculptures, Nachshon’s initial impression of the frequency and closeness of his interactions with his child shifted, and he gained another perspective. His experience of the gap in paternal representations and his affective reaction to it elicited a questioning and wondering stance that encouraged controlled mentalization.

**FIGURE 3:**
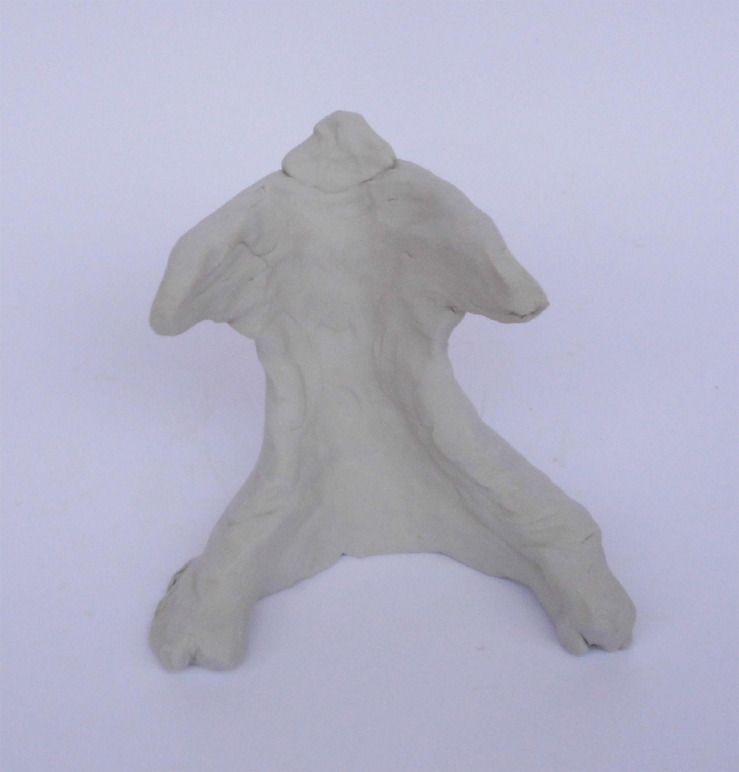
Roi’s sculpture.

Three fathers responded with an apparent mentalizing stance to questions specifically focused on their thoughts and feelings during the sculpting process. Their thinking process was at times explorative and revealed curiosity, but then they stopped and gave a very simple, matter-of-fact, external explanation in response to their own questions. For example, Roi, father of Shira, a 39-month-old girl, expressed wonder while observing his sculpture. Roi sculpted out of the lump of clay one figure that he described as an abstract representation of a hug, or a yearning for a hug (Figure 3). When Roi was asked to describe the sculpting process, he appeared to try to understand the meaning of his sculpting choices:

Roi: A thought that comes up now is that… I think you didn’t limit me in the… I mean, in the figures… You didn’t say to me “you need to make yourself,” and I’m trying to think why I didn’t sculpt the child…

However, he quickly resolved his wonder with a very matter-of-fact answer:

Roi: I don’t think it’s a special reason. Just because I thought that he… that one needs… that what… what he represents here, that is, because it is about me, so {gesturing with his hands toward the sculpture} (yes) but my identity as a father, so…

Roi first said he was “trying to think,” appearing to self-mentalize about his motives and choices when sculpting. At the same time, he seemed to be mentalizing about the interviewer’s expectations or judgments. After dismissing the possibility of any “special reason” and using the word “just”—a common indicator of the closing down of a mentalizing process (Bateman and Fonagy, 2013)—he then answered his own question by attributing it to his choice of subject matter. The interviewer sensed and respected his reluctance to discuss the topic further, despite the opportunity to examine it from different perspectives. This example may indicate the power of clay work to potentially stimulate a deeper, mentalization-based discourse about implicit parental representations; however, it also signals the need for caution when using this task in a research setting.

In Nachshon’s case, the physical dynamics and transformative character of the clay appear to have fostered non-verbalized (controlled) mentalization during the sculpting process and subsequent explicit reflection. For Roi, reflection on his sculpting choices elicited wondering and provided opportunities for controlled mentalizing. In both cases, the affective experience evoked also generated switches between controlled and automatic mentalization (lapsing into a less-reflective, implicit state of mind). However, they appeared to engage in controlled, reflective mentalizing as implied by their choice of subject matter and use of symbolism and metaphors (e.g., the dilemma of protecting or encouraging independence), as will be discussed in the following sections.

### Observing the Sculpture/Sculpting Process Revealed Gaps in Paternal Representations

As in Roi’s case, the sculpture served as a visual reflective object for six of the fathers (54%). Through visual descriptions of the sculptures, gaps between wishes, fantasies, and real actions divulged, which in turn enabled a fresh look at implicit paternal representations. In some cases, this led to a more explicit, contemplating look at themselves as fathers. Three of the fathers offered examples of new insights about themselves, using words such as “now that I look at it…” and “now… it came to my mind.” Table 3 summarizes the events of potential non-verbalized mentalization processes, identified by integrating the thoughts fathers shared about their presculpting planning phases with the main themes appearing in the interviews and the sculptures themselves.

**TABLE 3:** Indications of potential non-verbalized mentalization processes.

Preplanning nature of the sculpture**	Number of participants	Assumed mentalization process that was not explicitly verbalized while sculpting**
Thought about a specific situation and then positioned the figures accordingly.	4	Pondering a wish for closeness, the child’s needs versus the father’s needs, or a gap between a wish and reality.
Sculpted initial symbolic figures, and then physically moved the figures closer and further from each other, actively exploring their main dilemma.	3	The physical positioning of the figures as a metaphor for emotional concerns (distancing vs. protecting).
Used an abstract object as a metaphor to describe the concern he had decided to deal with at the start.	2	Pondering a wish for closeness or/and a gap between a wish and reality.
Planned and sculpted two symbolic/metaphoric objects but added an additional sculpted object—as new associations came to mind.	2	Use of objects as metaphors and symbols for either a wish or memory of specific interactions with the child (What do I want my child to learn? What did it mean to my child and to me?)

**As indicated, verbally, by the father either pre- or postsculpting.

**FIGURE 4:**
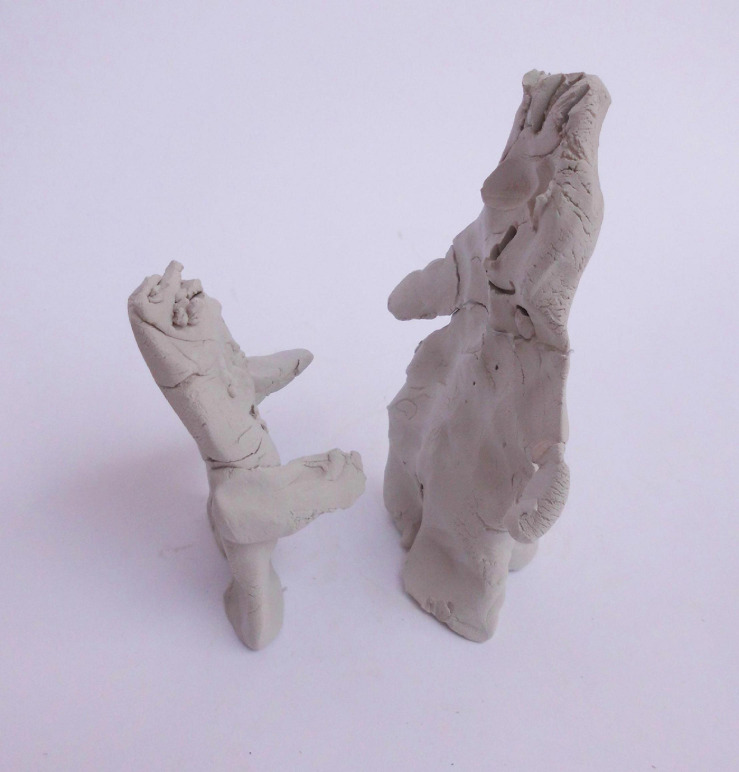
Tamir’s sculpture.

For example, Tamir, father of Ram, a 24-month-old boy, quickly sculpted father and son figures standing face-to-face but not touching each other. He explained that the child was reaching up so his father could lift him and hug him (Figure 4). The situation that appeared in the sculpture, in Tamir’s words, was his return home from work, when his excited son comes toward him and looks up at him in amazement. In his initial description of the sculpture, he observed the father as having room to contain the child. He expressed pride and joy in his sculpture and the way his son looks up to him. However, as the interview unfolded, Tamir mentioned that at first, he wanted to sculpt a bicycle, but then he discarded the idea. When asked if he wanted to add anything, he said (while reflecting on the sculpture):

Tamir: I don’t have a problem with it being in the Museum, but […] like, I didn’t invest much effort into it, that’s the truth. It’s a little, maybe also some kind of criticism toward me, that I don’t […] maybe don’t even invest enough in the relationship. (or that you are tired, coming in the evening and…) First of all, I arrive, I’m there, I’m not [..] don’t disappear. But […] it could be that not […] Why didn’t I make a bicycle, actually? It would have taken five more minutes? Maybe it would have looked terrible, but it doesn’t matter, but uh… (yes) Like, it could be that sometimes I also really give up, uh… I give up on myself, and don’t come back early enough to take him with the bicycle or…

Sparked at first by the question “What do you see?,” Tamir’s reflections on the visual outcome of the sculpture developed slowly, throughout the interview, in relation to the content of the interview itself. The sense of pride and containment described at first gradually turned into self-criticism and the desire to be more present and invested during the time spent with his son. Verbally reflecting upon this implicit paternal representation appears to have evoked a deeper mentalization process with potential for more elaboration. Similarly, other fathers related to gaps between wishes and reality—observations with the potential to unveil implicit paternal representations and facilitate mentalization. However, not all fathers elaborated on these issues verbally; some used the process of sculpting and the sculptures themselves.

### The Preplanning of the Sculptures Sparked Non-verbal Exploration of Metaphors and Symbolism

The prevalent appearance of symbolism and metaphors—before and during the sculpting process—indicate a reflective stance. These symbolic reflections included instances of perspective-taking (considering the child’s perspective, for example) and comparing ideals or desires to reality. Nachshon, mentioned above, appeared to be mentalizing while sculpting and using the distance between the father and child figures as a metaphor for the inner conflict between his desire to be a close and present father and his occasional feelings of mental and physical distance.

**FIGURE 5:**
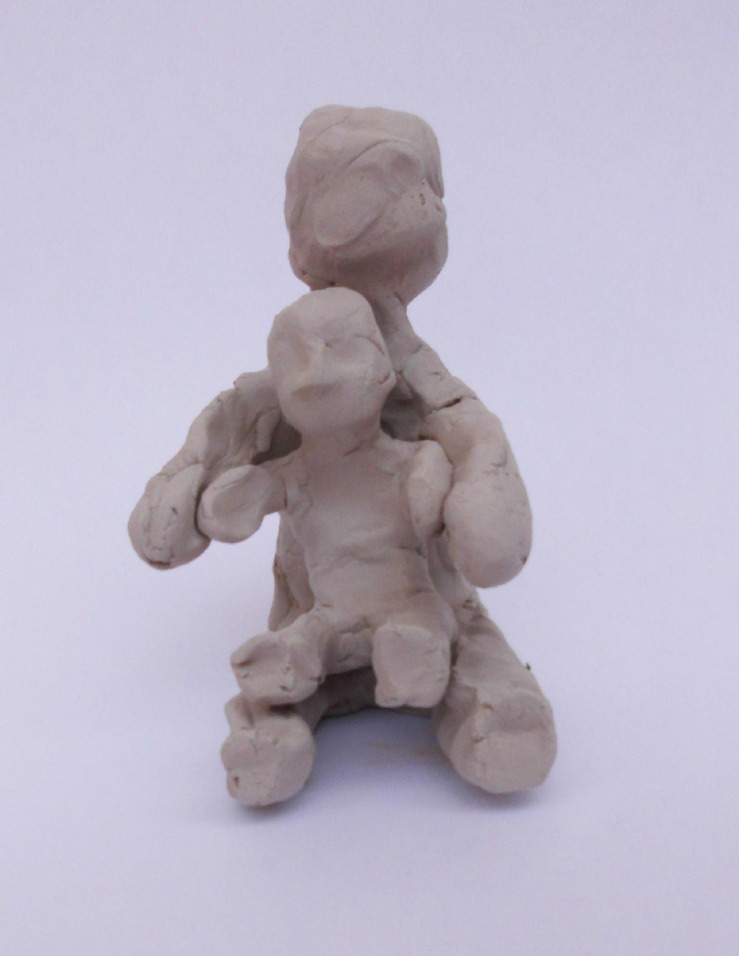
Shalom’s sculpture.

Another example is Shalom, father of Yoel, his 30-month-old boy. Shalom sculpted a sitting father and a similar-looking son sitting in his lap (Figure 5). While sculpting the child figure, he expressed wonder about the clay process, using the word “shaping,” which can signify both the physical shaping of clay and the educational and emotional shaping of his child and himself:

Shalom: it’s interesting {whispering} [..], trying to shape, shape yourself, shape someone else (yeah). {sharp change of tone – into a louder, clearer voice} But this is what I meant, like, that it’s hard for me to, like [..] to [..] to have [……] {voice fades away}, (to open up?) yeah, to open up, or to like, to, like, sort of just let it flow. There’s no flow in me.

The contemplation of the sculpting process may be seen here as a form of wonder, or a shift from conscious and concrete issues (wanting to sculpt the child sitting in his lap) to discovery of new insights. He later used the similar metaphor of “uterine thing” (in his words), reinforcing the associations of both holding and creating. Switching this metaphorical train of thought into personal observations on his difficulty to “open up” and his tendency to “overanalyze” at times, he went into a short discussion of his parents’ similar tendencies and his wish to give his son a different experience. In other words, metaphorical thinking during the sculpting process led to observations on self and implicit parental representations, which in turn led to reflection on intergenerational transmissions.

All 11 fathers described having had an idea in mind of the sculpture’s concept, main theme, or figure positioning before starting to sculpt (see Table 3). Since many of them focused on symbolizing preconceived ideas during the sculpting process, one might assume that the fathers were contemplating and possibly mentalizing explicitly, though not out loud, about the meaning of these ideas. While we do not know this for sure, the metaphoric and conceptual aspects of the final sculptures as described by the fathers indicate the appearance of controlled mentalizing that was essential for their creation. The main themes chosen for the sculptures include dilemmas between protecting versus encouraging independence and gap between wish about fatherhood and reality. Visual representation of these themes requires a certain amount of reflection, such as recollecting specific interactions with the child, and self-questioning in a reflective way, for example: “What do I wish for as a father?”; “In which moments do I feel a connection to my child?”; “What does my child feel in this situation?” (in the three cases, the fathers smiled to themselves as they drew a smile on the child’s figure); and so forth.

To summarize, Shalom and Nachshon’s examples may shed light on the mentalizing that they, and other fathers, experienced during the sculpting process. Using symbols and metaphors, the fathers appeared to mentalize without verbally describing all their explicit reflections. The fathers described a train of thoughts that began with planning the sculpture and continued through the process itself, ending in observation of the outcome. The verbally articulated observations included expressions of curiosity and wonder in response to reflections about the sculpting process and sculptures. Gaps between implicit and explicit paternal representations were also observed. These post-sculpting descriptions appear to be the tip of the iceberg of implicit thoughts and processes that could potentially be explored further in the context of long-term clinical interventions.

## Discussion

The main aim of this study was to identify what preceded the appearance of controlled mentalization among fathers of toddlers during a clay figure-sculpting task. An additional aim was to explore the nature of these processes underlying controlled paternal mentalization. The findings suggest that, within this context, controlled mentalizing may appear in two different channels: verbal mentalization, which appeared primarily during the interview in relation to sculpting-process-related questions; and non-verbal mentalization, which was deciphered through the conceptual nature of symbols and metaphors among the sculptures. Non-verbal mentalization requires verbal elaboration to be observed, since “we cannot translate the language of the unconscious into consciousness without lending it words from our conscious realm” (Klein, 1957, p. 180, footnote.). In the transition into words, the use of the sculpture and sculpting process as a reflective tool revealed gaps between verbal and non-verbal information, as will be elaborated below.

### Switches Between Implicit and Explicit Mentalization

There is no clear-cut borderline between implicit and explicit mentalization (Verfaille, 2016), and since their different forms are interconnected, they are at times difficult to distinguish. When observed, fluctuations between automatic and controlled and between cognitive and affective mentalizing appeared primarily with the sharing of deeply personal and/or emotional information, or when an apparent conflict between intentions and results was noticed by the father. These switches may, therefore, be a result of high arousal, i.e., feeling overwhelmed, ashamed, embarrassed (due to performance anxiety, for example), threatened, or confronted with out-group members (Luyten and Fonagy, 2015). They may have resulted from the absence of a therapy-alliance-related safe setting (Frank and Frank, 1991). The use of art materials may have contributed to high arousal resulting in mentalization switches. Attachment dimensions appear to be linked to responses to artistic media, with specific gender differences, pointing to a link between men’s attachment anxiety and negative attitudes toward art materials that provide a controlled experience, such as markers (Snir et al., 2017). While clay tends to evoke much arousal, it may also enable a controlled experience (Moon, 2010). While the fathers did not verbalize this, the preplanned nature of the sculptures and their topics could also be connected to a need to focus on a safe and familiar subject. Avoiding the experience of playing with clay without a preconceived plan reduces the risk of diving into the unconsciousness and uncovering unknown and potentially uncomfortable material (Sholt and Gavron, 2006).

### The Facilitation of Paternal Mentalization

The findings appear to strengthen Bat Or’s (2010) observations that the elicitation of wondering through the sculpting process, the transformative character of the clay, and the use of the sculpture as a visual reflective object all facilitate mentalization. The clay work’s elicitation of implicit childhood memories was not explored in this study, because its appearance, in most cases, was very subtle. Implicit knowledge is difficult to put into words (Verfaille, 2016), and it is possible that performance anxiety, indicated by mentalizing switches, hindered affective mentalization (Fonagy and Luyten, 2009) and, thus, the sharing of childhood memories. However, it is precisely these switches that allow us to observe, *in vivo*, implicit paternal representations in the form of activation of early, deeply rooted attachment systems and coping methods as an automatic reaction to stress (Luyten and Fonagy, 2015). The fathers rarely initiated discussion of intergenerational transmissions. One possible explanation could be that the fathers’ implicit and embodied parental representations are more maternal than paternal oriented and therefore the embodied attachment-related sensations are more abstract and difficult to access and represent. This is consistent with Zarfaty-Asherov’s (2007) findings that children of both sexes tend to represent the father figure less than the mother figure when using the story completion task. This explanation is reinforced by many of the descriptions, during the interviews, of participants’ fathers being distant, less physically close, and busy providing for the family. Based on his clinical experience in Israel, Samana (2016) expresses a need for a more active and goal-oriented paternal therapeutic stance. In art therapy, Kramer’s (1979) third hand concept encourages the therapist to assist with materials and remain active as an artist within the therapy. It may be that in a clinical and safe setting, if the therapist/researcher took a more active role, such as creating an artistic response during a mentalization-based session (Havsteen-Franklin and Altamirano, 2015), this could be especially appropriate for fathers, allowing for a gradual move from implicit to explicit mentalizing through non-verbal communication and on to verbal reflection on the art and process.

#### Non-verbal Explicit Mentalization and Verbal Implicit Mentalization

Within the sculpting processes, the appearance of controlled mentalization insinuated by symbols and metaphors was noteworthy. The fathers were presumably putting a lot of cognitive efforts into visually defining and expressing their declared preconceived topics and concerns. Despite this, the nature of the clay work appeared to have affected the controlled planning of the sculpture’s symbolic aspects, through its sensory-motor, difficult to control, transformative attributes. Even without intention, our minds and bodies are constantly in motion and changing—this dynamic flow constitutes forms of vitality (Stern, 2010). Implicit parental representations arose through these dynamic experiences, eliciting, in some cases, wondering and visually induced reflection. As a result, complex alternation between automatic and controlled mentalizing emerged; some non-verbal data expressed controlled reflective mentalizing (complex, planned, symbolism, and metaphor) and some verbal data expressed automatic, implicit mentalizing (giving reflexive, externally based explanations for discrepancies, for example). Mentalizing through non-verbal communication is possible even when the core capacities of the language system are dysfunctional (Willems et al., 2011).

In these specific sculpting processes, non-verbal mentalization does not necessarily imply not using language, since explicit thoughts may require the use of language as well (Newton and de Villiers, 2007). Some of the mentalization that was present during the sculpting process may have been explicit to the fathers themselves without being articulated to the interviewer. Stern (2009) defined the non-verbal-reflexive domain as an experience that is refigured into implicit, corporal-based knowledge but is not language-based. Verbal reflexivity, by contrast, consists of body-based experiences refigured into language. Based on this theory, the non-verbalized explicit mentalization noted in this study may fall somewhere in between these two domains, as a refiguration into bodily experience as well as language, in the form of thoughts or visual symbols. An example of a non-verbal switch in mentalization can be found in Nachshon’s (Figure 2) sculpting process, in which he automatically puts his son’s figure at a certain distance (applying implicit, quick mentalization about his closeness to his son) and then, switching to controlled reflection about himself as a father, places his son’s figure closer to him. By describing his thought process, Nachshon revealed this initially non-verbal mentalization. The use of a sculpture and a sculpting process makes the noting of links, biases, and discrepancies more tangible and harder to dismiss while still allowing the fathers to escape into silence or to gain an understanding or contemplative stance that does not always require words. The viewing of the sculpture during the postsculpting interview also enables embodied simulation, as the artwork evokes mirror mechanisms in the brain that in turn trigger bodily feelings, mimicking the physical experiences and sensations involved in the process of sculpting (Gallese, 2017). Essentially, while the fathers are asked to cognitively re-experience the sculpting process, they may also be re-experiencing it on an embodied level, adding an implicit, self-mentalizing dimension to the whole experience.

### The Use of Symbolism and Metaphor: Embodied Versus Abstract and Distanced From Bodily Sensations

Not all conceptual metaphors are manifested in the words of a language; some are manifested in gesture and art, for example, but they may be “secondarily expressed through language and other symbolic means” (Lakoff and Johnson, 1999, p. 57). The capacity for basic mentalizing precedes the capacity to play with symbols (Havsteen-Franklin, 2016a). Accordingly, to create a metaphor, the patient cannot be overly aroused or underaroused (Havsteen-Franklin, 2016b), as this would hinder the ability to mentalize and, therefore, to think metaphorically (Barnett, 2008). Therefore, to create a sculpture with explicitly declared symbolism or metaphors (as articulated by the fathers), it is probable that an underlying mentalizing process has taken place.

*Primary metaphors* are acquired automatically and unconsciously, based on embodied experiences, while *universal conceptual metaphors* are learned without being innate (Lakoff and Johnson, 1999). One might assume that men acquire gender-specific metaphors based on their embodied experience as well as learning them from others; however, both processes are influenced by societal gender norms. Goss (2008) suggests that “although attributes, behaviors and perceptions free-float and are shared between men and women, there are nevertheless some key differences in how men and women generally experience life, and how they embody and present these shared elements” (p. 147).

When compared with the mothers’ sculptures in Bat Or’s (2010) study, an explicit symbolic and metaphorical inclination was more dominant among the fathers’ sculptures.^3^ The fathers’ sculptures related primarily to objects, animals, “mini-me” figures (e.g., Figures 2, 4, 5) or depictions of an ideal father–son activity, whereas the metaphors offered by mothers in Bat Or’s (2010) study were primarily representations or extensions of their own body, for example holding or containing. The mothers created mother–child figures intuitively and developed the sculpture themes gradually during the sculpting process. Their experiences may have been linked to affective empathy, associated with automatic and embodied mentalizing (Sabbagh, 2004), connected to bodily sensations, and only subsequently integrated into cognitive knowledge (Luyten and Fonagy, 2015). While the mothers were drawn to this sensation-related body-based process, the fathers’ less body-based experience of the child (not having experienced pregnancy, birth, or breastfeeding) may have led to a more cognitive, controlled mentalizing during the sculpting process, as they attempted to process information about self and other in a more abstract and symbolic way.

This hypothesis is strengthened by the data about the fathers’ paternity leave (Table 1)—on average, they spent less time than the mothers being physically close to the child in the first months of life. Additionally, some fathers explained that their physical closeness to the child was partially dependent on the mother’s ability to “let go,” encouraging the father to take an active role in raising the child. This is consistent with clinical observations that mothers play a pivotal role in supporting or undermining the father–child relationship, reinforcing the concept of maternal “gatekeeping” (Fagan and Barnett, 2003). The fathers described excitement and anticipation toward the growing language and motoric abilities of the child that would allow them to get closer to their child through learning and playing, after not having been as physically close to the child as the mothers were through pregnancy and breastfeeding.

In a study among Australian parents of 12-month-old children, higher paternal mentalizing capacities were associated with parenting self-efficacy, family functioning, and fathering role perceptions (Cooke et al., 2017). Differences between maternal and paternal mentalizing capacities were found, suggesting a slightly higher mentalizing capacity among mothers. However, fathers who spent more time with their children on weekends showed higher mentalizing capacities.

Based on his clinical observations and lived experiences as a male art therapist, Vick (2007) asserts that men in therapy connect emotionally through physical as well as mental actions such as choosing, reflecting, and evaluating without words, and they eventually verbalize the psychological dimensions of these experiences. Based on Pollack (1998), he describes the male client’s need for a period of quiet to process emotions before sharing them. This may explain why verbal instances of mentalizing among the fathers appeared primarily after the sculpting process, especially toward the end of the interviews. Many of the fathers appeared to require time to think and/or needed action (sculpting) before elaborating about emotions. Thus, the use of art may have started a process of explicit mentalizing through actions and experience (Havsteen-Franklin, 2016b; Verfaille, 2016).

### Suggestions for Use in Clinical Practice

This task could potentially be expanded to include fathers in different clinical circumstances. The unique processing time and active exploration involved in the sculpting process seems to foster paternal mentalization. In the appropriate clinical environment, it might enable the father to gain a more profound understanding of his relationship with his child and to reflect upon implicit paternal representations.

This method could be used by clinicians in two potential contexts: (1) in the course of therapy, either as an additional tool to assess the father’s mentalizing capacities or as a parental intervention at a critical point in the course of his child’s therapy (e.g., Bat Or, 2012); and (2) as important information that contributes to a deeper understanding of paternal mentalization and of fathers’ subjective experiences within a mentalization-based art therapy intervention. Art therapists might be able to use this research to examine their attitudes toward fathers’ mentalization in their clinical practice.

Encouraging fathers to use metaphors as well as asking direct questions about their subjective experiences in relation to fatherhood may be an effective way of bypassing their initial inhibitions or concerns about taking an active role in their child’s therapy and participating in parental education.

### Limitations and Suggestions for Future Research

Due to the small sample size and specific demographic and sociocultural background of the participating fathers, it is hard to draw generalized conclusions from this study. Adding questions to the preinterview questionnaires, such as how actively involved each father was in his child’s daily routine and the mothers’ perception of the fathers’ involvement, might have contributed to a broader understanding of the context.

Another important point to consider is the influence of the geopolitical events in the region at the time of the interviews, namely, the Israel-Gaza conflict in the summer of 2014. Some of the fathers were soldiers in the reserves, had experienced war in the past, and knew they might be called up. Others lived in areas that were at risk of being hit by rockets. Both experiencing and inflicting trauma are associated with impaired mentalizing capacities (Allen et al., 2008). Parents and children risk the collapse of their mentalization abilities during trauma (Cohen, 2009), but parents are also usually preoccupied with providing a safe and healing setting for their children. Stress and awareness of death may have impeded mentalization. At the same time, a re-examining of basic assumptions and reordering of priorities may also have occurred (Allen, 2006), which potentially enhanced a reflective stance. It is very difficult to assess the exact effect these events may have had on the interviews and findings, as the fathers were not asked directly about this topic. Further investigation into the effects of national life-threatening situations on paternal mentalization may be of special relevance due to the COVID-19 pandemic and its implications on parent–child interactions (e.g., Shorer and Leibovich, 2020).

Reactivity of the participants toward the interviewer, specifically concerning her being a woman, may have had an impact on the father’s mentalization as well as the symbols and metaphors that arose. The influence of the interviewer’s opposite gender (Berger, 2015) was taken into account and referred to in an explicit question in most of the interviews. Some of the fathers mentalized in response to these explicit questions in relation to their wives and the researcher. These findings were not included in this paper and merit further, deeper investigation.

Additional quantitative measures, such as a control group and the coding and classification of RF—e.g., the Parental Reflective Functioning Questionnaire (Luyten et al., 2017) or PDI (Slade et al., 2004), might have contributed to a deeper understanding of the link between the fathers’ RF scores and the appearance of mentalization during the interviews and sculpting processes. In addition, a larger sample, as well as a comparison between groups of fathers from different cultural backgrounds, is proposed for future research. A specific comparison between fathers from countries that do or do not encourage paternity leave as a policy might be merited. A comparison between this art-based intervention and a verbal-only based intervention would also enhance our understanding of non-verbal mentalization and, specifically, the differences between implicit and explicit mentalization as they appear within or in relation to clay work. It is possible that other forms of art, such as drawing or creating an installation with found objects, would be as effective at eliciting paternal mentalization. This would be an additional angle that could be examined and developed further—with special attention to the unique differences between the characteristics of each material and how experiencing them may affect mentalization.

## Conclusion

Indications of controlled mentalizing among fathers in the study appeared mainly during the postsculpting interview as responses to the elicitation of wondering about the sculpting process, the transformative character of the clay, and the use of the sculpture as a visual reflective object. The use of symbolism and metaphor appears to have enriched and expanded the fathers’ multilayered capacities to express mentalization about their child, and themselves as parents. Detected differences between embodied representations and abstract representations of symbolism and metaphor may indicate that clay uniquely contributes to the study of implicit parental mentalization.

## Data Availability Statement

The datasets generated for this study are available on request to the corresponding author.

## Ethics Statement

The studies involving human participants were reviewed and approved by the Ethics Committee of Haifa University. Approval number 147/14. Participants signed written informed-consent forms to participate in this study, in accordance with the Declaration of Helsinki. Written informed consent was obtained from the individual(s) for the publication of any potentially identifiable images or data included in this article.

## Author Contributions

MB initially developed the task and semi-structured interview for her Ph.D. research project and focused on a sample of mothers. NG adapted the task and semi-structured interview for the purpose of her research project and focused on a sample of fathers, for her MA thesis dissertation. NG conducted and video-taped the clay tasks and individual interviews, and analyzed both verbal and visual data. MB supervised the research project. Both authors contributed to the article and approved the submitted version.

## Conflict of Interest

The authors declare that the research was conducted in the absence of any commercial or financial relationships that could be construed as a potential conflict of interest.

## Footnotes

1 More details can be found in the Supplementary Table 8.

2 See Supplementary Table 8.

3 See Supplementary Material online for more information: Supplementary Tables 4–7.

## References

[B1] Allen J. (2006). Oklahoma city ten years later: positive psychology, transactional analysis, and the transformation of trauma from a terrorist attack. *Trans. Anal. J.* 36 120–133. 10.1177/036215370603600205

[B2] Allen J. G. Fonagey P. Bateman A. W. (2008). *Mentalizing in Clinical Practice*. Washington, DC: American Psychiatric Publishing, Inc.

[B3] Baradon T. Fonagy P. Bland K. Lenard K. Sleed M. (2008). New beginnings- an experience-based programme addressing the attachment relationship between mothers and their babies in prisons. *J. Child Psychother.* 34 240–258. 10.1080/00754170802208065

[B4] Barnett A. J. (2008). Transformations in treatment: sublimatory implications of an interdisciplinary hypothesis on the metaphoric processing of emotional experience. *Psychoanal. Rev.* 95 79–106. 10.1521/prev.2008.95.1.79 18315467

[B5] Bat Or M. (2010). Clay sculpting of mother and child figures encourages mentalization. *Arts Psychother.* 37 319–327. 10.1016/j.aip.2010.05.007

[B6] Bat Or M. (2012). Non-verbal representations of maternal holding of preschoolers. *Arts Psychother.* 39 117–125. 10.1016/j.aip.2012.02.005

[B7] Bat Or M. Grenimann Bauch N. (2017). Paternal representations and contemporary parenthood themes through a clay figure-sculpting task among fathers of toddlers. *Arts Psychother.* 56 19–29. 10.1016/j.aip.2017.07.004

[B8] Bateman A. Fonagy P. (2013). Mentalization-base treatment. *Psychoanal. Inq.* 33 595–613. 10.1080/07351690.2013.835170 26157198 PMC4467231

[B9] Behrends A. Müller S. Dziobek I. (2012). Moving in and out of synchrony: a concept for a new intervention fostering empathy through interactional movement and dance. *Arts Psychother.* 39 107–116. 10.1016/j.aip.2012.02.003

[B10] Benbassat N. Priel B. (2015). Why is fathers’ reflective function important? *Psychoanal. Psychol.* 32 1–22. 10.1037/a0038022

[B11] Berger R. (2015). Now I see it, now I don’t: researcher’s position and reflexivity in qualitative research. *Qual. Res.* 15 219–234. 10.1177/1468794112468475

[B12] Betensky M. G. (1995). *What Do You See? Phenomenology of Therapeutic Art Expression*. London: Jessica Kingsley.

[B13] Bowlby J. (1969). *Attachment and Loss: Vol. 1. Attachment*. London: Hogarth Press.

[B14] Braun V. Clarke V. (2006). Using thematic analysis in psychology. *Qual. Res. Psychol.* 3 77–101. 10.1191/1478088706qp063oa 32100154

[B15] Bureau J.-F. Martin J. Yurkowski K. Schmiedel S. Quan J. Moss E. (2016). Correlates of child–father and child–mother attachment in the preschool years. *Attach. Hum. Dev.* 19 130–150. 10.1080/14616734.2016.1263350 27899058

[B16] Carson T. R. Samura D. (eds) (1997). *Action Research as a Living Practice*. New York, NY: Peter Lang.

[B17] Cohen E. (2009). “Parenting in the throes of traumatic events,” in *Treating Traumatized Children: Risk, Resilience and Recovery*, eds Brom D. Pat-Horenczyk R. Ford J. D. (East Sussex: Routledge), 72–84.

[B18] Cooke D. Priddis L. Luyten P. Kendall G. Cavanagh R. (2017). Paternal and maternal reflective functioning in the western australian peel child health study. *Infant Men. Health J.* 38 561–574. 10.1002/imhj.21664 28833359

[B19] Dollberg D. Feldman R. Keren M. (2010). Maternal representations, infant psychiatric status, and mother–child relationship in clinic-referred and non-referred infants. *Eur. Child Adolesc. Psychiatry* 19 25–36. 10.1007/s00787-009-0036-5 19543936

[B20] Elbrecht C. (2012). *Trauma Healing at the Clay Field: A Sensorimotor Approach to Art Therapy*. London: Jessica Kingsley.

[B21] Erickson F. (1992). “The interference between ethnography and microanalysis,” in *The Handbook of Qualitative Research in Education*, eds LeCompte M. D. Millroy W. L. Preissle J. (San Diego, CA: Academic Press), 201–225.

[B22] Esbjørn B. H. Pedersen S. H. Daniel S. I. F. Hald H. H. Holm J. M. Steele H. (2013). Anxiety levels in clinically referred children and their parents: examining the unique influence of self-reported attachment styles and interview-based reflective functioning in mothers and fathers. *Br. J. Clin. Psychol.* 52 394–407. 10.1111/bjc.12024 24117912

[B23] Fagan J. Barnett M. (2003). The relationship between maternal gatekeeping, paternal competence, mothers’ attitudes about the father role, and father engagement. *J. Fam. Issues* 24 24–39. 10.1177/0192513X03256397

[B24] Fonagy P. Allison E. (2012). “What is mentalization? The concept and its foundations in developmental research,” in *Minding the Child: Mentalization-Based Interventions With Children, Young People and Their Families*, eds Midgley N. Vrouva I. (Routledge: East Sussex), 11–34.

[B25] Fonagy P. Gergely G. Jurist E. Target M. (2002). *Affect Regulation, Mentalization and the Development of the Self*. New York, NY: Other Press.

[B26] Fonagy P. Luyten P. (2009). A developmental, mentalization-based approach to the understanding and treatment of borderline personality disorder. *Dev. Psychopathol.* 21 1355–1381. 10.1017/S0954579409990198 19825272

[B27] Fonagy P. Target M. (2007). The rooting of the mind in the body: new links between attachment theory and psychoanalytic thought. *J. Am. Psychoanal. Assoc.* 55 411–456. 10.1177/0003065107055002050 17601099

[B28] Fonagy P. Target M. Steele H. Steele M. (1998). *Reflective-Functioning Manual. Version 5. For Application to Adult Attachment Interviews*. London: University College London, 161–162.

[B29] Frank J. D. Frank J. B. (1991). *Persuasion and Healing: A Comparative Study of Psychotherapy*, 3rd Edn. Baltimore: Johns Hopkins University Press.

[B30] Gallese V. (2017). “The empathic body in experimental aesthetics – embodied simulation and art,” in *Empathy. Palgrave Studies in the Theory and History of Psychology*, eds Lux V. Weigel S. (London: Palgrave Macmillan).

[B31] Goss P. (2008). “Envisaging animus: an angry face in the consulting room,” in *Dreaming the Myth Onwards: New Directions in Jungian Therapy and Thought*, ed. Huskinson L. (East Sussex: Routledge).

[B32] Havsteen-Franklin D. (2016a). “Mentalization-based arts psychotherapy,” in *Approaches to Art Therapy Theory and Technique*, 3rd Edn, ed. Rubin J. A. (New York, NY: Routledge), 144–163.

[B33] Havsteen-Franklin D. (2016b). *When is a Metaphor? Art Psychotherapy and the Formation of the Creative Relationship Metaphor*. Doctoral dissertation, University of Essex, Essex.

[B34] Havsteen-Franklin D. Altamirano J. C. (2015). Containing the uncontainable: responsive art making in art therapy as a method to facilitate mentalization. *Int. J. Art Ther.* 20 54–65. 10.1080/17454832.2015.1023322

[B35] Haßelbeck J. H. (2014). *Paternal Mentalization Ability and Infant Development: Attachment, Father-Child Play, Emotional Regulation. [German – Väterliche Mentalisierungsfähigkeit und Kleinkindentwicklung: Bindung, Vater-Kind-Spiel, Emotionsregulation (Diplomarbeit in Psychologie)]*. Dissertation thesis in psychology, University of Vienna, Vienna.

[B36] Henley D. (2002). *Clayworks in Art Therapy: Playing the Sacred Circle*. Philadelphia: Jessica Kingsley.

[B37] Hinz L. D. (2009). *Expressive Therapies Continuum: A Framework for Using Art in Therapy*. New York, NY: Taylor & Francis Group.

[B38] Klein M. (1957). “Envy and Gratitude,” in *The Writing of Melanie Klein, Volume 3. Envy and Gratitude and Other Works*, ed. Money-Kyrle R. E. (New York: The Free Press), 176–235.

[B39] Kramer E. (1979). *Childhood and Art Therapy*. New York, NY: Schocken Books.

[B40] Lakoff G. Johnson M. (1999). *Philosophy in the Flesh: The Embodied Mind and its Challenge to Western thought*. New York, NY: Basic Books.

[B41] Lawrence P. J. Davies B. Ramchandani P. G. (2012). Using video feedback to improve early father-infant interaction: a pilot study. *Clin. Child Psychol. Psychiatry* 18 61–71. 10.1177/1359104512437210 22434935 PMC3834733

[B42] Luca M. (2009). *Embodied Research and Grounded Theory*. Cardiff: University of Wales.

[B43] Lucassen N. Tharner A. Van IJzendoorn M. H. Bakermans-Kranenburg M. J. Volling B. L. Verhulst F. C. (2011). The association between paternal sensitivity and infant–father attachment security: a meta-analysis of three decades of research. *J. Fam. Psychol.* 25 986–992. 10.1037/a0025855 22004434

[B44] Lundy B. L. (2013). Paternal and maternal mind-mindedness and preschoolers’ theory of mind: the mediating role of interactional attunement. *Soc. Dev.* 22 58–74. 10.1111/sode.12009

[B45] Lusebrink V. Hinz L. (2016). “The expressive therapies continuum as a framework in the treatment of trauma,” in *Art Therapy, Trauma, and Neuroscience*, ed. King J. L. (New York, NY: Routledge), 42–66.

[B46] Luyten P. Fonagy P. (2015). The neurobiology of mentalizing. *Personal. Disord.* 6 366–379. 10.1037/per0000117 26436580

[B47] Luyten P. Mayes L. C. Nijssens L. Fonagy P. (2017). The parental reflective functioning questionnaire: development and preliminary validation. *PLoS One* 12:e0176218. 10.1371/journal.pone.0176218 28472162 PMC5417431

[B48] Lyons-Ruth K. (1998). Implicit relational knowing: its role in development and psychoanalytic treatment. *Infant Ment. Health J.* 19 282–289.

[B49] Mascaro J. S. Hackett P. D. Gouzoules H. Lori A. Rilling J. K. (2013). Behavioral and genetic correlations of the neural response to infant crying among human fathers. *Sci. Direct. Brain Res.* 1580 78–101. 10.1093/scan/nst166 PMC4221211 24336349

[B50] Moon C. H. (2010). *Materials & Media in Art Therapy*. New York, NY: Routledge.

[B51] Nan J. K. M. Ho R. T. H. (2017). Effects of clay art therapy on adults outpatients with major depressive disorder: a randomized controlled trial. *J. Affect. Disord.* 217 237–245. 10.1016/j.jad.2017.04.013 28433887

[B52] Newton A. M. de Villiers J. G. (2007). While talking thinking. *Psychol. Sci.* 18 574–579. 10.1111/j.1467-9280.2007.01942.x 17614864

[B53] Oxford Dictionary. (2019). *Oxford Dictionaries, s.v. “Symbol,”*. Available online at: https://en.oxforddictionaries.com/definition/symbol (accessed January 2, 2019).

[B54] Patton M. Q. (2002). *Qualitative Research & Evaluation Methods*, 3rd Edn. Thousand Oaks, CA: Sage.

[B55] Pleck J. H. (2010). “Paternal involvement: revised conceptualization and theoretical linkages with child outcomes,” in *The Role of the Father in Child Development*, 5th Edn, ed. Lamb M. E. (Hoboken, NJ: Wiley), 58–93.

[B56] Pollack W. (1998). *Real Boys: Rescuing Our Sons from the Myths of Boyhood*. New York, NY: Holt.

[B57] Ricoeur P. (1981). *Hermeneutics and the Human Sciences: Essays on Language, Action and Interpretation*. Cambridge: Cambridge University Press.

[B58] Rubin J. A. (1999). *Art Therapy – An Introduction*. Philadelphia: Brunner/Mazel.

[B59] Sabbagh M. A. (2004). Understanding orbitofrontal contributions to theory-of-mind reasoning: implications for autism. *Brain Cogn.* 55 209–219. 10.1016/j.bandc.2003.04.002 15134854

[B60] Samana R. (2016). Thinking ‘outside of the womb’ (Hebrew). *Discussions* 31 7–18.

[B61] Sarkadi A. Kristiansson R. Oberklaid F. Bremberg S. (2007). Fathers’ involvement and children’s developmental outcomes: a systematic review of longitudinal studies. *Acta Paediatr.* 97 153–158. 10.1111/j.1651-2227.2007.00572.x 18052995

[B62] Schechter D. S. Coots T. Zeanah C. H. Davies M. Coates S. W. Trabka K. A. (2005). Maternal mental representations of the child in an inner-city clinical sample: violence-related posttraumatic stress and reflective functioning. *Attach. Hum. Dev.* 7 313–331. 10.1080/14616730500246011 16210242

[B63] Schiborr J. Lotzin A. Romer G. Schulte-Markwort M. Ramsauer B. (2013). Child-focused maternal mentalization: a systematic review of measurement tools from birth to three. *Measurement* 46 2492–2509. 10.1016/j.measurement.2013.05.007

[B64] Scott K. L. Crooks C. V. (2007). Preliminary evaluation of an intervention program for maltreating fathers. *Brief Treat. Crisis Interv.* 7 224–238. 10.1093/brief-treatment/mhm007

[B65] Shai D. Belsky J. (2011). When words just won’t do: introducing parental embodied mentalizing. *Child Dev. Perspect.* 5 173–180. 10.1111/j.1750-8606.2011.00181.x

[B66] Sharp C. Fonagy P. (2008). The parent’s capacity to treat the child as a psychological agent: constructs, measures and implications for developmental psychopathology: topic review. *Soc. Dev.* 17 737–754. 10.1111/j.1467-9507.2007.00457.x

[B67] Sholt M. Gavron T. (2006). Therapeutic qualities of clay-work in art-therapy and psychotherapy: a review. *Art Ther.* 23 66–72. 10.1080/07421656.2006.10129647

[B68] Shorer M. Leibovich L. (2020). Young children’s emotional stress reactions during the COVID-19 outbreak and their associations with parental emotion regulation and parental playfulness. *Early Child Dev. Care* 1–11. 10.1080/03004430.2020.1806830 [Epub ahead of print].

[B69] Slade A. (2005). Parental reflective functioning: an introduction. *Attach. Hum. Dev.* 7 269–281. 10.1080/14616730500245906 16210239

[B70] Slade A. (2007). Reflective parenting programs: theory and development. *Psychoanal. Inq.* 26 640–657. 10.1080/07351690701310698

[B71] Slade A. Bernbach E. Grienenberger J. Levy D. W. Locker A. (2004). *The Parent Development Interview and the Pregnancy Interview: Manuals for Scoring*. New Haven, CT: City College of New York.

[B72] Snir S. Regev D. Shaashua Y. H. (2017). Relationships between attachment avoidance and anxiety and responses to art materials. *Art Ther.* 34 20–28. 10.1080/07421656.2016.1270139

[B73] Söderström K. Skårderud F. (2013). The good, the bad, and the invisible father: phenomenological study of fatherhood in men with substance use disorder. *Fathering* 11 31–51. 10.3149/fth.1101.31

[B74] Souter-Anderson L. (2010). *Touching Clay: Touching What? The Use of Clay in Therapy*. Dorset: Archive Publishing.

[B75] Steele H. Steele M. (2008). “On the origins of reflective functioning,” in *Mentalization: Theoretical Considerations, Research Findings, and Clinical Implications*, ed. Busch F. (New York, NY: Taylor & Francis), 133–158.

[B76] Stern D. N. (2009). Pre-reflexive experience and its passage to reflexive experience. a developmental view. *J. Conscious. Stud.* 16 307–331.

[B77] Stern D. N. (2010). *Forms of Vitality: Exploring Dynamic Experience in Psychology and the Arts, Psychotherapy and Development*. Oxford: Oxford University Press.

[B78] Suchman N. E. DeCoste C. L. McMahon T. J. Dalton R. Mayes L. C. Borelli J. (2017). Mothering from the inside out: results of a second randomized clinical trial testing a mentalization-based intervention for mothers in addiction treatment. *Dev. Psychopathol.* 29 617–636. 10.1017/S0954579417000220 28401850 PMC5407293

[B79] Tharner A. Altman F. H. Væver M. (2016). Fathers’ perceptions of caregiving in childhood and current mentalizing with their preschool children. *Nord. Psychol.* 68 176–193. 10.1080/19012276.2015.1125302

[B80] Verfaille M. (2016). *Mentalizing in Arts Therapies*. London: Karnac.

[B81] Vick R. M. (2007). The boy is father to the man: introduction to the special issue on men in art therapy. *Art Ther.* 24 2–3. 10.1080/07421656.2007.10129363

[B82] Wheelock J. (1990). Families, self-respect and the irrelevance of “rational economic man” in a postindustrial society. *J. Behav. Econ.* 19 221–236. 10.1016/0090-5720(90)90012-V

[B83] Willems R. M. Benn Y. Hagoort P. Toni I. Varley R. (2011). Communicating without a functioning language system: implications for the role of language in mentalizing. *Neuropsychologia* 49 3130–3135. 10.1016/j.neuropsychologia.2011.07.023 21810434

[B84] Zarfaty-Asherov Y. (2007). *Six-Year-Olds’ Representations of the Father Figure: Gender Differences and the Relationship to Paternal Parenting Attitudes and Beliefs*. MA. Thesis, University of Haifa, Haifa.

