# Supplementary material for: Exploring Paternal Mentalization Among Fathers of Toddlers Through a Clay-Sculpting Task
Source: Front Psychol. 2021 Mar 2;12:518480. doi: 10.3389/fpsyg.2021.518480 (PMC7960673; doi:10.3389/fpsyg.2021.518480)
Supplement: Supplementary file 1 [file Data_Sheet_1.PDF]

Supplementary Material I – *Sculptures of parent-toddler relationships; comparisons between mother and father samples*

The following tables provide additional information regarding comparisons between the sample of 11 fathers who participated in the current study and a sample of 24 mothers who underwent the same task in a previous study. The data about the mother sample was retrieved from Bat Or (2010, 2012)[1] and raw material received by permission from the author; including images, videos, and transcripts. Due to the difference in sample size, each phenomenon is mentioned in percentages – to demonstrate what proportion of the sample it is. The tables compare between main phenomenological and verbal themes that arose in both samples. These themes are relevant to the article's discussion about differences between mother and father samples who underwent the same clay sculpting task. Specifically, they demonstrate an inclination within the fathers' sample towards less-body-based metaphors. The following are descriptions of each comparison table:

I. **Levels of abstraction and realism of the sculptures (with images)** – based on the fathers' and mothers' descriptions of the sculptures as well as on visual phenomena.

II. **The separation between sculpted objects** – demonstrating to what extent the parent and child figures (or the different non-human elements) in each sculpture are perceived as separate or connected. Each sample had its scale of separation relevant to its themes (defined, for example, by whether the parent started sculpting with one lump of clay or two, what the parent said about the figures holding each other, etc.). In addition, the fathers' sample was assessed according to the mothers' sample scale of separation (since the mothers had few sculptures in which the sculptured figures were not touching each other at all, while the father samples had many separately sculpted and placed figures).

III. **Levels of holding and verbal themes** – demonstrating to what extent the parent figure is shown or described as holding the child figure, and to what extent do these correspond to the verbal themes of holding raised by each sample set. Two separate scales appear in the table – one scale within each sample (for example, higher levels of holding within the father sample were based more on the fathers' verbal explanations about the scenario than on the visual phenomena) and one scale in which the fathers' sample is compared to the mothers' holding scale (which focused primarily on visual phenomena).

IV. **Movement and grounding** – in this case only one scale was used for both samples. Visual phenomena, coupled with verbal descriptions by the parents, was used to define to what extent the sculpture appears to be "in movement" (whether the sculpted figures appears to easily be able to move – by the appearance of legs, for example) and to what extent it appears to be "grounded" (whether the sculpted figure appear sturdy and/or "stuck" in place). These themes, similar to the previous themes of separation and holding, are deemed relevant to demonstrate a stronger inclination among the mothers to sculpt the child as a baby in their arms compared to an inclination among the fathers to sculpt separate figures described as moving.

---

[1] Bat Or, M. (2010). Clay sculpting of mother and child figures encourages mentalization. Arts in Psychotherapy, 37(4), 319–327. https://doi.org/10.1016/j.aip.2010.05.007
Bat Or, M. (2012). Non-verbal representations of maternal holding of pre-schoolers. The Arts in Psychotherapy, 39, 117–125.

Supplementary Material II – *Sculptures of parent-toddler relationships; verbal and nonverbal mentalization, additional demographic data*

The following tables allow the reader a closer view of the verbal mentalization instances found in the interviews and the indications of non-verbalized mentalization processes. Additional demographic and background information about the participants is also presented:

V. **Additional demographic data:** this table brings additional data collected about the fathers, including their level of education, artistic experience and religious background.

VI. **The Sculpting Task and Interview:** The full list of tasks and questions used within this research (as developed originally by Dr. Michal Bat Or[2]).

1 Based on definitions of RF types in Fonagy, P., Target, M., Steele, H., & Steele, M. (1998). Reflective-functioning manual, version 5.0, for application to adult attachment interviews. London: University College London, 161-2.

2 Bat Or, M. (2010). Clay sculpting of mother and child figures encourages mentalization. Arts in Psychotherapy, 37(4), 319–327. https://doi.org/10.1016/j.aip.2010.05.007

1.1 List of tables:

I. **Table 4.** Sculptures by fathers versus sculptures by mothers – from abstract to realistic.

II. **Table 5.** Sculptures by fathers versus sculptures by mothers – the separation between sculpted objects.

III. **Table 6.** Sculptures by fathers versus sculptures by mothers – levels of holding and main verbal themes.

IV. **Table 7.** Sculptures by fathers versus sculptures by mothers – movement and grounding.

V. **Table 8.** Additional demographic data

VI. **The Sculpting Task and Interview**

Table 4. Sculptures by fathers versus sculptures by mothers – from abstract to realistic.

Level of abstraction/ realism	Sculptures by fathers	Sculptures by Mothers
**Abstract**	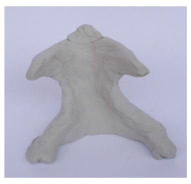 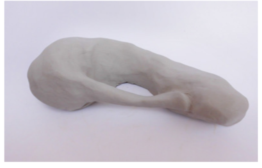 18.18% of the fathers' sample	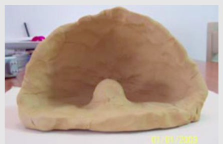 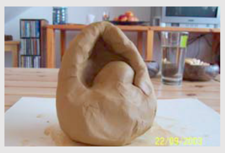 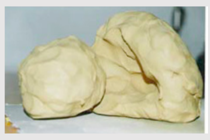 12.5% of the mothers' sample
**Semi-abstract** (simplified forms of two figures – resembling human or partially-human/ fantasy forms)	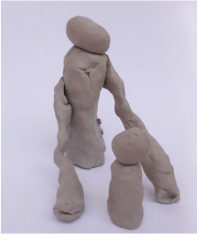 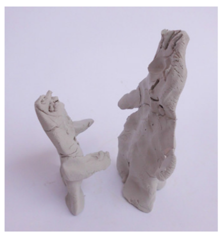 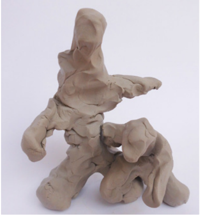 27.27% of the fathers' sample	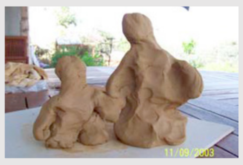 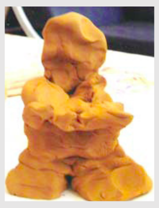 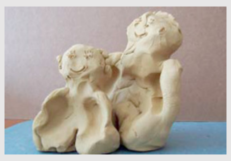 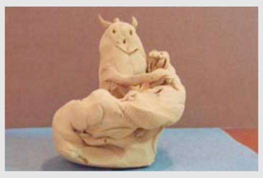 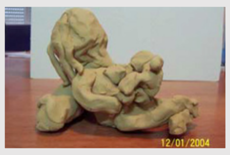 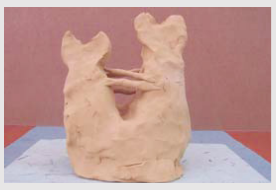 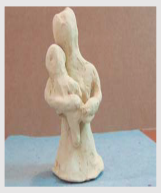 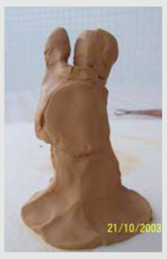 33.33% of the mothers' sample
**Metaphorical representations** (Explicitly non-human figures)	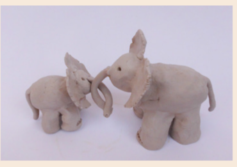 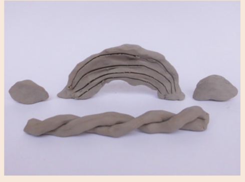 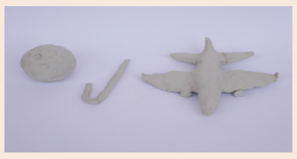 27.27% of the fathers' sample	0% of the mothers' sample
**Human figures with non-human (metaphorical) extensions of their body**	0% of the fathers' sample	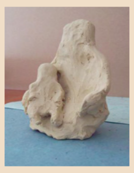 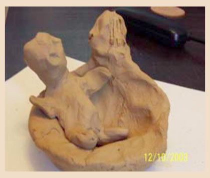 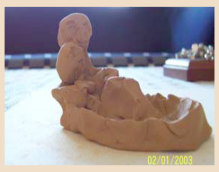 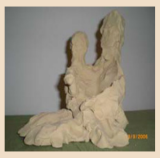 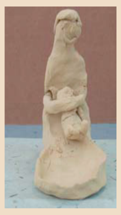 20.83% of the mothers' sample
**Realistic Figures** (two figures explicitly resembling humans, with detailed features)	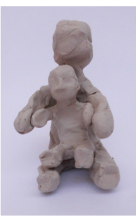 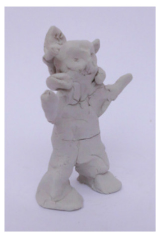 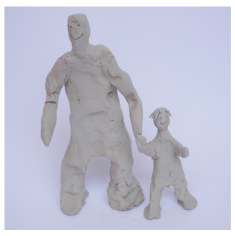 27.27% of the fathers' sample	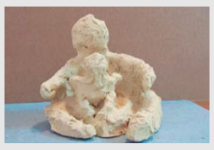 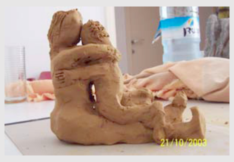 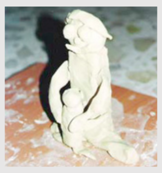 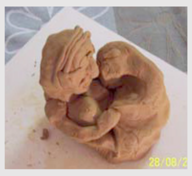 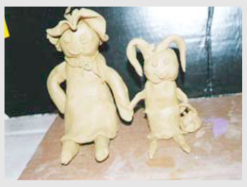 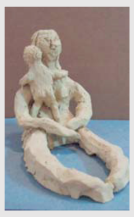 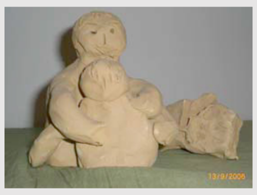 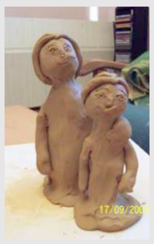 33.33% of the mothers' sample

Table 5. Sculptures by fathers versus sculptures by mothers – the separation between sculpted objects.

Number of sculpted objects				Separation (between sculpted figures) *Separate scales per study sample*			Separation – comparative scales *When fathers' sculptures are judged according to the original mothers' sculpture-separation scale*		
**Study sample**	**Number of Participants**	**Percentage of study sample**	**Objects**	**Number of**	**Percentage of study sample**	**Separation level**	**Number o Participants**	**Percentage of study sample**	**Separation level**
**Sample 1: mothers**	14	58.33%	Two	9	37.5%	High **	9	37.5%	High
	10	42.66%	One *	10	42.66%	Partial	10	42.66%	Partial
				5	20.83%	Low	5	20.83%	Low
**Sample 2: fathers**	7	63.63%	Two	2	18.18%	Abstract/ high***	10	90.90%	High
	2	18.18%	One	2	18.18%	High	1	9.09%	Low
	1	9.09%	Three	5	45.45%	Medium			
	1	9.09%	Four	2	18.18%	Abstract/ Low ****			

* Two figures/ representational objects merged into one

** Medium within the fathers' scale

*** Objects rather than figures - more than two separate objects

**** One-piece sculpture (one created from two lumps and merged, and one created from one lump)

Table 6. Sculptures by fathers versus sculptures by mothers – levels of holding and main verbal themes.

	**Holding** *(nonverbal data – separate scales for mother and father study samples)*			**Holding – comparative scales** *(when fathers' sculptures are judged according to the original mothers' sculptures scale)*			**Main verbal Themes**		
Study sample	#	%	holding level*	#	%	holding level	#	%	Separation level
**Sample 1: mothers**	7	29.16%	Active holding	7	29.16%	Active holding**	7	29.16%	Giving protection and adapted holding
	6	25%	Partially active holding	6	25%	Partially active holding	6	25%	Container theme: combining protection and encouragement for autonomy
	5	20.83%	Passive holding	5	20.83%	Passive holding	5	20.83%	Supporting autonomy vs. distancing anxiety
	5	20.83%	No holding	5	20.83%	No holding	5	20.83%	A gap between a symbiotic wish and the child's growing autonomy
**Sample 2: fathers**	3	27.27%	Relatively close holding	2	18.18%	Active holding**	3	27.27%	Closeness (hug, warmth, containment, intertwining)
	1	9.09%	Partially close holding	2	18.18%	Partially active holding	2	18.18%	Approaching to be close (reaching for a hug)
	3	27.27%	Distant/ symbolic holding	–	0%	Passive holding	3	27.27%	Protecting and encouraging
	4	36.36%	No holding***	7	63.63%	No holding ****	3	27.27%	Exploring together (playfulness, teaching, nourishing with knowledge)

* * Holding levels match mothers' verbal themes

** When defined as: parents arms bringing the child closer to the parent. In both cases of "active holding" among the father sample, this is an abstract sculpture and is based on their verbal explanations.

*** When defined as: no touching at all. Touching sculptures are defined as "Distant holding"

**** When defined as: no holding with or without supportive touching

# – Number of participants

% – Percentage of participants within study sample

Table 7. Sculptures by fathers versus sculptures by mothers – movement and grounding.

	**Movement** *(based on the father study's movement scale)*			**Grounding** *(standing base - legs or lump, and potential for movement)*		
Study sample	#	%	Movement level	#	%	Type of base
**Sample 1: mothers**	2	8.33%	A lot of movement (active walking, running, stretching arms)	4	16.66%	Figures standing on their legs (mother's legs are clearly defined)
	3	12.5%	Insinuation of a lot of movement (restricted or metaphoric)	4	16.66%	Figures sitting with legs (mother's legs are clearly defined)
	15	62.5%	Medium level movement (sitting with arm and upper body active)	4	16.66%	Figures standing without defined legs (standing on a lump-like base)
	4	16.66%	Slight hint of movement/ or no movement (abstract/ relatively still but flow and subject matter may indicate movement)	7	29.16%	Figures sitting without defined legs (in some cases: instead of legs there is a container/ surface)
				5	20.83%	Abstract/ or figures are merged
**Sample 2: fathers**	4	36.36%	A lot of movement (active walking, running, stretching arms)	5	45.45%	Figures standing on their legs (mother's legs are clearly defined)
	3	27.27%	Insinuation of a lot of movement (restricted or metaphoric)	2	18.18%	Figures sitting with legs (mother's legs are clearly defined)
	2	18.18%	Medium level movement (sitting with arm and upper body active)	1	9.09%	Figures standing without defined legs (standing on a lump-like base)
	2	18.18%	Slight hint of movement/ or no movement (abstract/ relatively still but flow and subject matter may indicate movement)	0	–	Figures sitting without defined legs (in some cases: instead of legs there is a container/ surface)
				3	27.27%	Abstract/ or figures are merged

# – Number of participants

% – Percentage of participants within study sample

Table 8: Additional Demographic Data

Father #	Religious status (all Jewish)	Academic background	Income	Years of marriage	Relationship satisfaction (1-5 scale[1])	Artistic (and clay sculpting) background	Sculpting time
**1**	Religious (moderate)	Second degree	Near average, without family assistance	3.3	4	No previous artistic background	3:39
**2**	Religious (moderate)	Doctoral candidate	Above average without family assistance	6	5	No previous artistic background	43:20
**3**	Secular	Doctoral candidate	Significantly above average due to inheritance	3	4	No previous artistic background	24:45
**4**	Traditional/ conservative	First degree	Above average with some family assistance	6	4	Other - degree in architecture in the arts department	22:50
**5**	Secular - atheist	Second degree	Above average without family assistance	5	5	No previous artistic background **a part from creating with wood**	21:06
**6**	Secular	Second degree	Above average with family assistance	4	5	No previous artistic background	3:55
**7**	Secular	First degree	Above average without family assistance	4	5	No previous artistic background	5:15
**8**	Secular	Second degree	Above average with family assistance	3.5	5	No previous artistic background	15:00
**9**	Traditional/ conservative	Second degree	Near average with family assistance	3	5	No previous artistic background	3:49
**10**	Secular	Full high school diploma	Above average with family assistance	3.5	3	No previous artistic background	8:18
**11**	Religious (moderate)	First degree	Near average with family assistance	4	4	No previous artistic background	2:41

[1] 1 = not satisfied at all, 5 = very satisfied

Paternal sculping task and sculpting interview

This is a sculpting task and semi-structured interview for parents, originally developed in Hebrew by Michal Bat Or (Sholt 2009). The questions originally addressed mothers and was later adapted by the authors to address fathers, as well. The task and interview are meant to be conducted individually, with one parent (without their child present).

Sculpting process

Preparation: the parent is given a single, round, 15cm x 15cm lump of clay, placed on a mobile wood surface with five wooden sculpting tools beside it.
In a warm-up stage, the interviewer conveys some basic instructions about working with clay to create three-dimensional sculptures. The participant is then told: "Please sculpt yourself with your child, or sculpt the relationship between you and your child." The participant is asked to create a three-dimensional sculpture. The sculpture can be in any style the participant wishes it to be (e.g., realistic, abstract, or mixed). The interviewer explains that there will be no aesthetic judgment of the sculpture. The sculpting processes are videotaped from the approximate perspective of the participant, focusing on her/ his hands.

During the sculpting process, participants may choose if to sculpt silently or speak to the interviewer. The sculpting process is not limited in time.

The Sculpting Interview

Following the sculpting process, the Sculpting Interview consists of several phases:

a. **Observation:** The created sculpture is observed carefully from all angles, with the interviewer rotating the sculpture slowly in front of the participant.

b. **Questions:**

1. What is the first thing you'd like to say now?
2. What do you see?
3. I will now ask you about the sculpting process: how was it for you, to touch the clay?
4. Please try to describe the sculpting process.
5. What are the thoughts and feelings you experienced during the sculpting process?
6. Do you remember whether you had obstacles or frustrations during the sculpting process? If you did, at what part of the process did they occur?
7. I will now ask you a few questions about the sculpture. What title would you find appropriate for your sculpture?
8. **If there are sculpted figures** – I will now ask about the figures in the sculpture:
9. If the mother/father figure in the sculpture could speak, what would she/ he say?
10. If the child figure in the sculpture could speak, what would she/ he say?
11. Try to construct a conversation between the figures.

12. If you had a chance, would you add/ change/ subtract anything from the sculpture?

13. Observing the sculpture now, is there anything you understand about yourself as a mother/ father, about your child, and/or about your relationship?

14. Is there anything in the sculpture that might depict an aspect of your relationship as a child with your own mother/father? If there is, please tell me about it, and if there isn't, how does it differ?

15. If you had been asked to sculpt yourself as a child with your mother/ father, imagine what the sculpture would look like and describe it.

16. If you had a choice, what would you like to do with this sculpture?

17. The meeting and interview will soon come to an end. How do you feel about parting with the sculpture that will stay here (with the interviewer)?

18. Is there anything else that did not come up in the interview, and you think is important to say?

19. What feelings are you leaving this meeting with?

C. Additional questions for fathers:

1. What are your thoughts about fatherhood? Do you feel your experiences of fatherhood differ from your father's experiences?
2. Do you feel that there is a difference in parenting tasks between you and your child's mother? Do you feel there are different societal expectations (for example – the way a teacher or therapist would speak to you versus the child's mother)?
1. Do you feel it makes a difference to you that I, the interviewer, am a woman? Would the interview feel different if conducted by a man?
